# The Evaluation of a Deep Learning Approach to Automatic Segmentation of Teeth and Shade Guides for Tooth Shade Matching Using the SAM2 Algorithm

**DOI:** 10.3390/bioengineering12090959

**Published:** 2025-09-06

**Authors:** KyeongHwan Han, JaeHyung Lim, Jin-Soo Ahn, Ki-Sun Lee

**Affiliations:** 1 Dental Research Institute and Department of Dental Biomaterials Science, School of Dentistry, Seoul National University, 101 Daehak-ro, Jongno-gu, Seoul 03080, Republic of Korea; dr.han2875@gmail.com; 2 Department of Oral & Maxillofacial Surgery, Korea University Ansan Hospital, Ansan si 15355, Gyeong-gi do, Republic of Korea; surgidenta@gmail.com; 3 Department of Prosthodontics, Korea University An-san Hospital, Ansan si 15355, Gyeong-gi do, Republic of Korea; 4 Medical Science Research Center, Ansan Hospital, Korea University College of Medicine, Ansan si 15355, Gyeong-gi do, Republic of Korea

**Keywords:** tooth segmentation, shade guide, SAM2, deep learning, dental AI

## Abstract

Accurate shade matching is essential in restorative and prosthetic dentistry yet remains difficult due to subjectivity in visual assessments. We develop and evaluate a deep learning approach for the simultaneous segmentation of natural teeth and shade guides in intraoral photographs using four fine-tuned variants of Segment Anything Model 2 (SAM2: tiny, small, base plus, and large) and a UNet baseline trained under the same protocol. The spatial performance was assessed using the Dice Similarity Coefficient (DSC), the Intersection over the Union (IoU), and the 95th-percentile Hausdorff distance normalized by the ground-truth equivalent diameter (HD95). The color consistency within masks was quantified by the coefficient of variation (CV) of the CIELAB components (L*, a*, b*). The perceptual color difference was measured using CIEDE2000 (ΔE00). On a held-out test set, all SAM2 variants achieved a high overlap accuracy; SAM2-large performed best (DSC: 0.987 ± 0.006; IoU: 0.975 ± 0.012; HD95: 1.25 ± 1.80%), followed by SAM2-small (0.987 ± 0.008; 0.974 ± 0.014; 2.96 ± 11.03%), SAM2-base plus (0.985 ± 0.011; 0.971 ± 0.021; 1.71 ± 3.28%), and SAM2-tiny (0.979 ± 0.015; 0.959 ± 0.028; 6.16 ± 11.17%). UNet reached a DSC = 0.972 ± 0.020, an IoU = 0.947 ± 0.035, and an HD95 = 6.54 ± 16.35%. The CV distributions for all of the prediction models closely matched the ground truth (e.g., GT L*: 0.164 ± 0.040; UNet: 0.144 ± 0.028; SAM2-small: 0.164 ± 0.038; SAM2-base plus: 0.162 ± 0.039). The full-mask ΔE00 was low across models, with the summary statistics reported as the median (mean ± SD): UNet: 0.325 (0.487 ± 0.364); SAM2-tiny: 0.162 (0.410 ± 0.665); SAM2-small: 0.078 (0.126 ± 0.166); SAM2-base plus: 0.072 (0.198 ± 0.417); SAM2-large: 0.065 (0.167 ± 0.257). These ΔE00 values lie well below the ≈1 just noticeable difference threshold on average, indicating close chromatic agreement between the predictions and annotations. Within a single dataset and training protocol, fine-tuned SAM2, especially its larger variants, provides robust spatial accuracy, boundary reliability, and color fidelity suitable for clinical shade-matching workflows, while UNet offers a competitive convolutional baseline. These results indicate technical feasibility rather than clinical validation; broader baselines and external, multi-center evaluations are needed to determine its suitability for routine shade-matching workflows.

## 1. Introduction

Achieving an accurate shade match between the natural dentition and dental restorations is a critical determinant of patient satisfaction and esthetic success in restorative, prosthetic, and esthetic dentistry [1,2]. Despite advances in dental materials and techniques, the final color of a restoration often becomes the most visible and most scrutinized component by patients [3,4]. Shade matching is traditionally performed using a visual comparison with standardized shade guides under clinical lighting conditions [2,5]. However, this method remains inherently subjective and is influenced by numerous human and environmental factors, including eye fatigue, clinician experience, lighting quality, emotional state, and even gender-based perceptual differences [3,6,7]. The translucency, thickness, and light interaction properties of enamel and dentin complicate visual estimation further, leading to considerable inter- and intra-observer variability [8].

To address these limitations, recent studies have explored digital and AI-assisted solutions for more objective and reproducible shade matching [2,6,7]. Nonetheless, most of the existing approaches focus on either tooth segmentation or a shade guide analysis independently, with limited consideration of their spatial relationship within the same clinical image. Furthermore, few efforts have been made to develop automatic segmentation models that jointly identify both the natural teeth and the shade guide while excluding irrelevant anatomical features such as the lips and gingiva—an essential step for robust shade-matching algorithms.

In this study, we propose and evaluate a deep learning approach using fine-tuned variants of Segment Anything Model 2 (SAM2)—specifically, the tiny, small, base plus, and large model configurations—to perform simultaneous semantic segmentation of both the teeth and shade guides in intraoral photographic images. SAM2 provides multiple configuration variants (tiny, small, base plus, and large), each utilizing different vision transformer (ViT) backbones with varying input resolutions, parameter sizes, and computational costs. As reported in the prior literature [9,10,11], these differences can significantly impact the segmentation accuracy, inference speed, and resource requirements, making model selection a critical aspect in domain-specific applications such as medical image analysis.

SAM2 is a foundation segmentation model developed to extend the zero-shot and promptable segmentation capabilities of its predecessor, SAM, while achieving higher accuracy and broader generalization across image domains. SAM2 leverages vision transformers (ViT) at various scales—such as tiny, small, base plus, and large—to support adaptive inference and fine-tuning across diverse data distributions. Unlike task-specific segmentation networks, SAM2 is trained on a massive corpus of natural images and supports flexible prompts such as points, boxes, and masks, enabling its application to previously unseen domains through minimal adaptation. While originally introduced for general-purpose image segmentation, recent studies have demonstrated its potential in medical image analysis tasks such as organ delineation in radiology, histopathology image segmentation, and surgical scene understanding, often outperforming conventional architectures when fine-tuned on domain-specific datasets [12,13,14]. This versatility makes SAM2 a promising backbone for intraoral image segmentation tasks, where precise delineation of the teeth and shade guides is critical and complicated by varying lighting, occlusions, and anatomical variability.

To contextualize SAM2 against a widely adopted convolutional architecture in medical image analysis, we also train and evaluate UNet as the baseline model under the same dataset and optimization settings. UNet employs a symmetric encoder–decoder with skip connections that fuse fine spatial details from shallow layers with context from deeper layers, enabling precise boundary localization and reliable segmentation of small structures. Its fully convolutional design supports arbitrary input sizes via an overlap tile strategy and efficient dense prediction. In the low-data regimes common in medical imaging, UNet trains stably with strong data augmentation and simple losses, converges quickly with modest computation, and offers fast inference with a relatively lightweight footprint. These properties make UNet a routine reference for accuracy and efficiency in biomedical segmentation studies [15,16,17,18].

Table 1 summarizes the architectural characteristics of the UNet baseline and the four SAM2 configurations (tiny, small, base plus, and large), including the backbone type, parameter count, input resolution, and indicative use cases.

To the best of our knowledge, this study is the first to systematically evaluate the comparative performance of multiple scales of SAM2—namely, tiny, small, base plus, and large. While SAM2 has recently garnered attention for its applicability in medical image segmentation, previous studies [19,20] have either conducted fine-tuning using only a single variant or have not explicitly indicated which variant was employed. However, no prior work has quantitatively examined and compared the segmentation performance across different SAM2 model scales within a clinical context.

By constructing a dataset comprising annotated intraoral photographs with co-located teeth and shade guides, this study provides a foundation for the development of AI systems capable of supporting clinicians in objective and reproducible shade assessments. It not only highlights the segmentation capabilities of SAM2 models across different scales but also opens new directions in AI-assisted esthetic dentistry, where an accurate and automated analysis of color-matching scenarios can significantly enhance both clinical decision-making and patient satisfaction.

## 2. Materials and Methods

### 2.1. The Experimental Design

**Figure 1:**
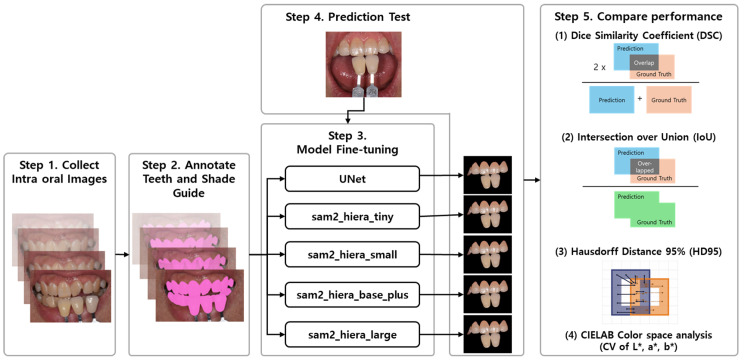
The experimental pipeline for SAM2-based tooth and shade guide segmentation. **Step 1:** Collect intraoral photographs containing the teeth and a physical shade guide. **Step 2:** Create pixel-wise ground-truth masks for the teeth and shade guide regions. **Step 3:** Fine-tune the baseline model (UNet) and the four SAM2 (Hiera) configurations (tiny, small, base plus, and large) **Step 4:** Run inference on the held-out test set. **Step 5:** Compare the performance using the DSC, IoU, and HD95 and a color-space analysis. The thumbnails illustrate representative examples; arrows indicate the data flow. The asterisk [*] denotes CIELAB coordinates: L* (lightness), a* (red–green), and b* (yellow–blue).

The experimental workflow comprised the following steps (Figure 1). First, a dataset was constructed by collecting intraoral photographs in which both the teeth and shade guides were co-located to enable shade determination. Second, manual annotation was performed to delineate the regions corresponding to the teeth and shade guides within each image. Third, four SAM2 variants—tiny, small, base plus, and large—were each fine-tuned on the prepared dataset, and a UNet baseline was trained under the same protocol for comparison. Fourth, the segmentation performance of all SAM2 configurations and the UNet baseline was quantitatively evaluated and compared. Finally, to assess the feasibility of shade determination using segmented outputs, the shade guide region was cropped from the original images and compared with the corresponding segmented regions by analyzing differences in the color-space range.

### 2.2. Data Collection

This retrospective study was conducted with approval from the Board of Ethics in Biomedical Research at Korea University Ansan Hospital (IRB No. 2023AS0034). A total of 500 intraoral photographs were randomly selected from the clinical records of patients who underwent prosthodontic treatment between 1 January 2018 and 31 December 2022 in the Department of Prosthodontics at Korea University Ansan Hospital. The selected images all included both visible natural teeth and a shade guide captured simultaneously under standard clinical lighting conditions for shade verification.

All photos were anonymized prior to processing. Only images containing permanent teeth and a shade guide were included. Images with excessive blurring, poor lighting, or occlusion that hindered visual segmentation were excluded.

### 2.3. Data Annotation

**Figure 2:**
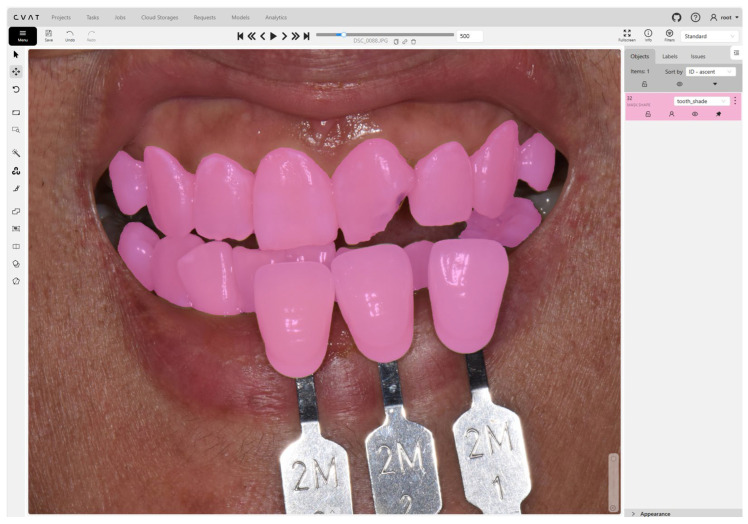
An example of annotation of the teeth and shade guides in an image containing both objects for tooth shade determination.

To prepare the dataset for segmentation, all 500 images were manually annotated using the Computer Vision Annotation Tool (CVAT, version 2.4.1, CVAT.ai Corp., Palo Alto, CA, USA). A representative annotation is shown in Figure 2. A board-certified prosthodontist with over 10 years of clinical experience performed the labeling. Each image was masked at the pixel level to delineate two distinct classes: (1) natural teeth and (2) the shade guide. Regions corresponding to irrelevant structures such as the lips, gingiva, or tongue were excluded from labeling to ensure focused segmentation accuracy. All annotations were reviewed and validated manually to ensure high-quality ground-truth labels for model training.

### 2.4. Model Training and Fine-Tuning

A fine-tuning pipeline was established for the four configurations of the Segment Anything Model 2 (SAM2)—tiny, small, base plus, and large—and, for comparison, a conventional UNet baseline. Each model was independently fine-tuned on the annotated dataset to develop customized semantic segmentation algorithms for the simultaneous identification of natural teeth and shade guides in intraoral photographs. To ensure a fair comparison, the training protocol, data splits, and evaluation procedures were harmonized across all SAM2 variants and the UNet baseline.

For each configuration, the image encoder and mask decoder modules were fine-tuned, while the prompt encoder was kept frozen to maintain the generalization capabilities, following established transfer learning protocols [1]. To ensure a controlled backbone comparison and to isolate architectural effects, we fixed the input resolution at 1024 × 1024 for all SAM2 variants for both training and evaluation. Although smaller variants (tiny/small) are often recommended at lower inputs for efficiency, the SAM2 encoder–decoder stack supports variable resolutions via positional embedding interpolation. Data augmentation techniques, including random changes in the brightness, contrast, and hue and spatial transformations, were applied to increase the model’s robustness and prevent overfitting. Specifically, brightness/contrast factors were sampled uniformly from [0.8, 1.2] (*p* = 0.8), the hue was shifted by ±10° in the HSV space (*p* = 0.5), and the spatial transforms comprised rotation (±12°, *p* = 0.8), scaling (0.90–1.10, *p* = 0.8), translation (±8% of the image size, *p* = 0.8), shear (±8°, *p* = 0.5), and horizontal flipping (*p* = 0.5; vertical flip *p* = 0). Bilinear interpolation was used on the images (nearest-neighbor masks), and they were resized to 1024 × 1024. Early stopping criteria were enforced based on the Dice Similarity Coefficient (DSC) on a held-out validation subset.

The dataset, consisting of 500 annotated intraoral images, was randomly divided into a training set (*n* = 400, 80%) and a testing set (*n* = 100, 20%), while ensuring class balance and no subject overlap between the two sets. To enable a fair performance comparison under identical conditions, the same training and testing datasets were used across all four SAM2 model configurations.

**The fine-tuning strategy and hyperparameters**: Training was performed using the AdamW optimizer (learning rate = 1 × 10^−4^; weight decay = 1 × 10^−4^) and a ReduceLROnPlateau scheduler (factor = 0.5; patience = 50; threshold = 1 × 10^−4^). Mixed precision training (PyTorch AMP) was employed, together with gradient accumulation (5 steps) and gradient clipping (max-norm = 1.0). The total number of training iterations was set to 10,000.

**Loss functions**: The segmentation objective was defined as a binary cross-entropy loss on the predicted masks, supplemented by a calibration term aligning the predicted mask confidence score with the observed Intersection over the Union (IoU). The total loss was expressed as

$$L=L_{seg}+0.05\times L_{score}$$

**Training monitoring and model selection**: The model performance was continuously monitored using the Dice Similarity Coefficient (DSC) and the IoU on a held-out validation subset. An exponential moving average of the IoU was used to track the convergence, and the model achieving the highest validation IoU was saved as the final checkpoint. Early stopping criteria were enforced to prevent overfitting.

All training procedures were conducted on a high-performance workstation equipped with an Intel^®^ Core™ i9-14900 CPU (Intel Corp., Santa Clara, CA, USA), 128 GB of DDR4 RAM, and an NVIDIA RTX 4090 GPU (NVIDIA Corp., Santa Clara, CA, USA) running Ubuntu 22.04 LTS. Model implementation and training were performed using Python 3.12 and PyTorch 2.0.

### 2.5. Evaluation

To comprehensively assess the performance of the segmentation models, both the spatial segmentation accuracy and color representation consistency were evaluated using the following criteria.

#### 2.5.1. Segmentation Performance

**Figure 3:**
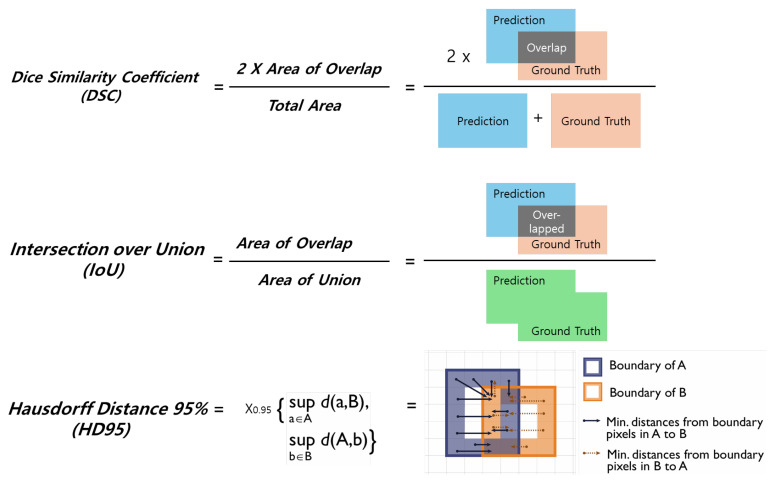
Visual explanation of the Dice Similarity Coefficient (DSC), Intersection over the Union (IoU), and Hausdorff Distance 95% (HD95) in the segmentation evaluation.

The accuracy of the predicted segmentation masks for the baseline UNet and the four SAM2 variants (tiny, small, base plus, and large) was assessed using three widely used segmentation metrics [21,22] (Figure 3):

**The Dice Similarity Coefficient (DSC)**: This metric quantifies the spatial overlap between the predicted mask and the manually annotated ground truth, ranging from 0 to 1. A value closer to 1.0 indicates a higher degree of agreement.

**The Intersection over the Union (IoU)**: Also referred to as the Jaccard index, the IoU is computed as the area of intersection divided by the area of union between the predicted and ground-truth masks. It serves as a robust indicator of the overall segmentation accuracy, especially in imbalanced regions.

**The Hausdorff Distance 95% (HD95)**: This is a boundary-sensitive metric that summarizes the 95th percentile of the bidirectional surface distances between the predicted and ground-truth mask boundaries. Compared with the maximum Hausdorff distance, HD95 reduces the sensitivity to isolated outliers while still reflecting large boundary errors. Lower values indicate closer boundary alignment. Because the images were acquired from heterogeneous cameras and the mm-per-pixel scale was unavailable, we report the HD95 in **pixels** and additionally provide a normalized form (HD95 divided by the ground-truth equivalent diameter, %) to mitigate scale variation across images.

All metrics were calculated separately for the segmented classes and macro-averaged to yield a single representative score for each image. The final performance scores were reported as the mean ± standard deviation across the 100-image test set.

#### 2.5.2. Color Uniformity and Perceptual Difference Evaluation

To evaluate the consistency of the color representation within the segmented regions, the coefficients of variation (CV) in the CIELAB color values—L*, a*, and b*—were computed for segmented regions [23]. The CV was defined as the ratio of the standard deviation to the mean of each color component within the respective region:

$$CV_{X}=\frac{\sigma_{X}}{\mu_{X}}\;\mathrm{for}\;X\in \{L^{*},a^{*},b^{*}\}$$

Lower CV values indicate greater homogeneity in the color distribution, suggesting more precise and stable segmentation of chromatically uniform areas. This evaluation was conducted for each image in the test set and averaged across the dataset to compare the color consistency across different SAM2 model configurations.

In addition to the CV analysis, perceived color differences were assessed using the CIEDE2000 (ΔE_00_) color difference formula [24,25]. For each image, the RGB values within the entire predicted mask and within the entire ground-truth mask were converted into CIELAB (D65/2°). We then computed the mean L*, a*, and b* over each full mask and measured a single ΔE00 between these two mean colors, using the standard parametric factors *k_L_ = k_C_ = k_H_ =* 1. This mean–full-mask comparison quantifies the global chromatic discrepancy between the predicted and annotated regions without restriction to the intersection pixels and is less sensitive to small boundary misalignments. Lower ΔE_00_ values indicate a closer perceptual match between the predicted and reference colors, providing a complementary measure of color accuracy beyond statistical uniformity.

## 3. Results

### 3.1. Segmentation Performance Evaluation

**Figure 4:**
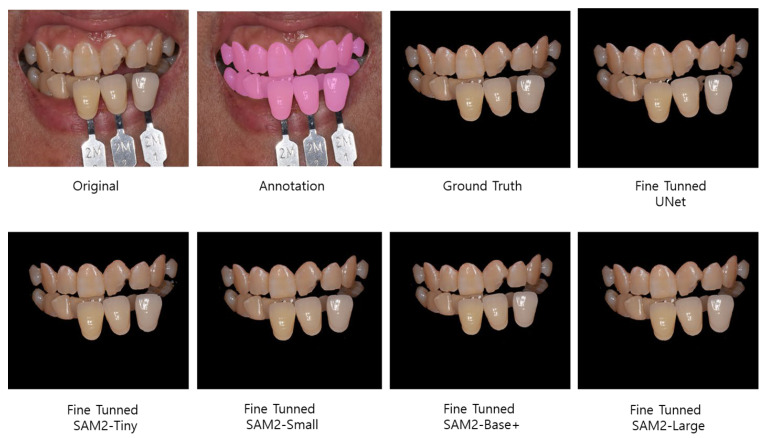
A representative example of the segmentation results for an intraoral photograph containing both teeth and shade guides. From left to right: An original image with shade tabs, manual annotation, annotated ground truth, and segmentation outputs generated by the baseline model (UNet) and four fine-tuned SAM2 model variants (sam2_tiny, sam2_small, sam2_base_plus, and sam2_large).

In this study, four variants of SAM2 (sam2_tiny, sam2_small, sam2_base_plus, sam2_large) were fine-tuned for simultaneous segmentation of the teeth and shade guides in intraoral photographs and compared with a UNet baseline. Figure 4 illustrates a representative qualitative result.

**Figure 5:**
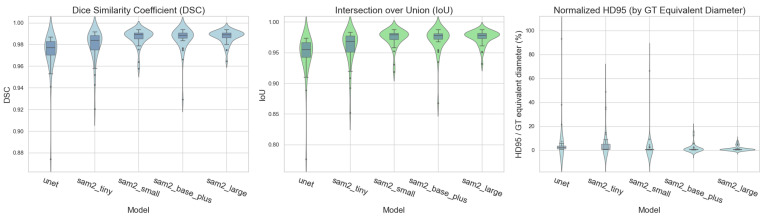
Violin plots with box overlays comparing the DSC (left), IoU (center), and normalized HD95 (right) across five models (UNet and four SAM2 variants) on the test dataset. Across all models, the DSC and IoU were high, whereas the HD95 (normalized) remained low, indicating a strong overlap and tight boundary alignment. Boxes denote the interquartile range with the median line; violins depict the per-image distributions. Higher is better for the DSC/IoU, while lower is better for the normalized HD95. The distributional patterns further show that larger-capacity variants tend to achieve a superior segmentation performance with tighter dispersion. The boxes represent the interquartile range (IQR), with the central line indicating the median; whiskers extend to 1.5 × IQR, and outliers are shown as points.

The segmentation performance of the UNet baseline model and the fine-tuned SAM2 models was evaluated using the DSC, IoU, and HD95 across a test set of 100 images. All models achieved a high overlap accuracy as follows; sam2_large performed best (DSC: 0.9871 ± 0.0060; IoU: 0.9746 ± 0.0116), followed by sam2_small (DSC: 0.9867 ± 0.0077; IoU: 0.9738 ± 0.0148), sam2_base_plus (DSC: 0.9852 ± 0.0113; IoU: 0.9710 ± 0.0212), sam2_tiny (DSC: 0.9790 ± 0.0150; IoU: 0.9592 ± 0.0279), and UNet (DSC: 0.972 ± 0.0195, IoU: 0.947 ± 0.0346). Consistent with the violin plot distributions (Figure 5), UNet showed a wider spread and more outliers, whereas sam2_large/sam2_small were tighter and more stable.

Complementing these overlap metrics, the boundary accuracy measured by the normalized HD95 (HD95 divided by the ground-truth equivalent diameter, %) exhibited the same ordering and highlighted differences in the boundary reliability—sam2_large = 1.25 ± 1.80%, sam2_base_plus = 1.71 ± 3.28%, sam2_small = 2.96 ± 11.03%, sam2_tiny = 6.16 ± 11.17%, and UNet = 6.54 ± 16.35% (lower is better)—indicating that larger variants not only overlap better but also align the boundaries more closely, while smaller models—particularly sam2_tiny and the baseline model UNet—show heavier-tailed errors for challenging cases.

### 3.2. Color Uniformity and Perceptual Difference Evaluation

#### 3.2.1. Color Uniformity Evaluation

**Figure 6:**
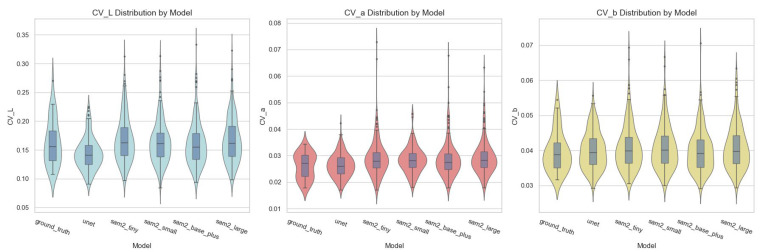
Violin plots with overlaid boxplots showing the distribution of the coefficient of variation (CV) values in the CIELAB color space across the ground truth, UNet, and four SAM2 variants: (**left**) L*, (**center**) a*, (**right**) b*. A lower CV indicates greater color uniformity within the segmented regions; all prediction models produce distributions closely matching the ground-truth variability. The boxes represent the interquartile range (IQR), with the central line indicating the median; whiskers extend to 1.5 × IQR, and outliers are shown as points.

The color uniformity of the segmented regions was evaluated by computing the coefficient of variation (CV) for each CIELAB component (L*, a*, b*). Figure 6 summarizes the distributions with violin plots and embedded boxplots. The ground-truth masks showed the lowest variability—L*: 0.164 ± 0.040; a*: 0.026 ± 0.005; b*: 0.040 ± 0.006—indicating a highly consistent color within the annotated regions. Among the prediction models, UNet produced CVs close to the ground truth (L*: 0.144 ± 0.028; a*: 0.026 ± 0.004; b*: 0.040 ± 0.005). The SAM2 variants also clustered near the ground-truth levels: sam2_base_plus (L*: 0.162 ± 0.039; a*: 0.029 ± 0.007; b*: 0.040 ± 0.006), sam2_small (L*: 0.164 ± 0.038; a*: 0.028 ± 0.005; b*: 0.041 ± 0.006), sam2_large (L*: 0.168 ± 0.040; a*: 0.029 ± 0.007; b*: 0.041 ± 0.006), and sam2_tiny (L*: 0.169 ± 0.040; a*: 0.029 ± 0.007; b*: 0.041 ± 0.007). Overall, both UNet and the SAM2 family markedly reduced the intra-mask chromatic variability and achieved CVs close to that of the ground-truth reference.

#### 3.2.2. Perceptual Color Difference Evaluation

**Figure 7:**
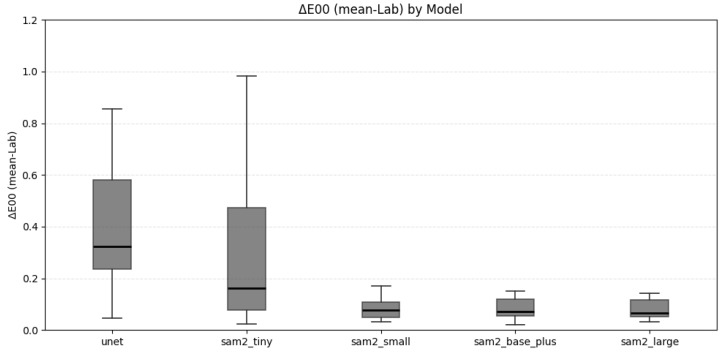
Boxplots of the per-image CIEDE2000 (ΔE_00_) computed as the distance between the mean CIELAB color inside each full prediction mask and that of the full ground-truth mask. Boxes show the interquartile range with the median line. Lower values indicate smaller perceptual color differences.

In addition to the CV analysis, perceptual color differences were evaluated using the ΔE_00_ metric. For each prediction–ground-truth pair, RGBs were converted into CIELAB, and we computed the mean L*, a*, and b* over the full predicted mask and over the full ground-truth mask. The CIEDE2000 distance between these two mean colors produced one ΔE_00_ per image. Figure 7 shows boxplots of these per-image ΔE_00_ values for each model on the test set. The summary statistics for each model, expressed as the median (mean ± SD), are as follows: UNet: 0.325 (0.487 ± 0.364); SAM2-tiny: 0.162 (0.410 ± 0.665); SAM2-small: 0.078 (0.126 ± 0.166); SAM2-base-plus: 0.072 (0.198 ± 0.417); and SAM2-large: 0.065 (0.167 ± 0.257). In aggregate, all models yield ΔE_00_ values well below the ≈1 just noticeable difference (JND) threshold, indicating close chromatic agreement between the predictions and annotated regions. The three larger SAM2 variants (sam2_small, sam2_base_plus, sam2_large) cluster around 0.1 ΔE_00_, while UNet and sam2_tiny show higher errors that are still below the JND.

## 4. Discussion

In this study, we systematically evaluated the segmentation performance and color uniformity of four fine-tuned SAM2 variants—tiny, small, base plus, and large—together with a UNet baseline on a clinically relevant task: simultaneous segmentation of the natural teeth and shade guides in intraoral images. To our knowledge, this is the first report to compare multiple SAM2 scales in a dental imaging context specifically oriented toward shade determination.

### 4.1. The Segmentation Performance

Across the test set (*n* = 100), all SAM2 variants achieved a high overlap accuracy, with SAM2-large showing the best overall performance (DSC: 0.987 ± 0.006; IoU: 0.975 ± 0.012). The general ordering for the overlap was large > small > base plus > tiny, with UNet trailing (DSC: 0.972 ± 0.020; IoU: 0.947 ± 0.035). The performance trend observed—large > small > base plus > tiny—suggests a positive correlation between the model scale and segmentation accuracy. In previous studies that utilized the earlier version of SAM—rather than SAM2—for medical imaging tasks, large-scale variants of the model were typically employed during fine-tuning [13,26]. Similarly, in the present study, the SAM2-large variant yielded the highest segmentation performance among the four configurations. However, the segmentation performance cannot be assumed to increase linearly with model scale. Notably, although the difference was not statistically significant, the SAM2-small model slightly outperformed the SAM2-base plus variant, suggesting that smaller-scale models may still achieve comparable or even superior results depending on the task and dataset characteristics. Consequently, the superiority of SAM2-large may be attributed to its higher model capacity and ability to retain detailed contextual information during fine-tuning. However, even the lighter models such as SAM2-small and SAM2-base plus showed a competitive performance, which is encouraging for resource-constrained deployment in clinical environments.

To complement the overlap metrics, we assessed the boundary reliability using the HD95. Because images were acquired with heterogeneous optics and no physical scale was available, we additionally report a normalized form of the HD95 divided by the ground-truth equivalent diameter (%). The normalized HD95 was consistent with the DSC/IoU ranking yet offered finer boundary discrimination, with SAM2-large demonstrating the highest performance (HD95: 1.25 ± 1.80%). Larger-capacity models concentrated in the lower single-digit percent range with tight dispersion, whereas the small and especially the tiny variant showed broader, heavy-tailed distributions. Thus, larger-capacity variants not only achieve a higher overlap but also align the boundaries more closely and more consistently, whereas the tiny (and to a lesser extent small) model shows heavy-tailed errors for challenging cases (e.g., glare, occlusions). These findings underscore that boundary-sensitive metrics capture clinically relevant aspects of mask quality—such as precise shade-tab and tooth margins—that may not be fully reflected by the DSC/IoU alone.

### 4.2. Color Uniformity and Implications for Shade Matching

An important contribution of this study is the evaluation of the color uniformity within segmented regions using the CV in the CIELAB components (L*, a*, b*). This aspect is rarely addressed in segmentation benchmarks but is particularly critical for tasks involving color-based diagnosis or prosthodontic shade matching [1,2,23,27].

The ground-truth annotations exhibited the lowest CVs, reflecting clean and consistent region definitions. All prediction models closely tracked these distributions. For example, the mean ± SD for L* CV was 0.161 ± 0.039 (SAM2-base plus), 0.164 ± 0.038 (SAM2-small), 0.168 ± 0.040 (SAM2-large), 0.169 ± 0.040 (SAM2-tiny), and 0.144 ± 0.028 (UNet), compared with 0.164 ± 0.040 for the ground truth; a* and b* showed similarly tight bands across models. These patterns indicate that once relevant regions are isolated, the resulting masks are chromatically homogeneous—an essential prerequisite for reliable shade assessment. Among the models, SAM2-base plus and SAM2-small most closely approximated the ground truth in terms of color uniformity. This suggests that despite minor differences in the boundary segmentation metrics, smaller models may preserve chromatic homogeneity better —potentially due to reduced overfitting or smoother mask boundaries. These findings underscore the importance of evaluating not only spatial accuracy but also functional relevance—in this case, the consistency of color representation critical for shade selection.

Complementing the CV analysis, we assessed perceived color differences using the CIEDE2000 (ΔE_00_) formula computed per image between the mean CIELAB color of the full predicted mask and that of the corresponding ground-truth mask (Figure 7). The resulting distributions are compact and remain below the ≈1 JND threshold for all models. Consistent with the summary statistics reported above, the three larger SAM2 variants concentrate near approximately 0.1 ΔE_00_ with tight interquartile ranges, while UNet and sam2_tiny show higher medians and broader dispersion with occasional outliers. Nevertheless, their values still fall below the JND threshold, indicating that the predicted colors are perceptually close to the references. By definition, ΔE_00_ = 0 denotes identical colors; values around ≈1 are barely perceptible, ≈2–3 are clearly perceptible, and ≥5 are obvious [24,25,28]. In this context, the clustering near the JND suggests that the predicted regions are, on average, perceptually very close to their references. Together with the CV findings, this supports that the models not only suppress background-driven inhomogeneity but also preserve perceptual color accuracy in the segmented areas, a desirable property for shade-relevant applications.

### 4.3. Clinical and Technical Implications

Fine-tuned SAM2 models, particularly the larger configurations, provide robust and reproducible masks with tight boundaries and chromatically uniform interiors, supporting objective shade-matching workflows that reduce subjectivity in clinical practice. From a deployment standpoint, SAM2-small and SAM2-base plus offer attractive accuracy–efficiency trade-offs for chairside use, while UNet remains a practical convolutional reference when computational budgets are constrained. The mean full-mask ΔE00 formulation also aligns with how clinicians compare overall tooth and shade-tab appearance, offering a perceptually meaningful, task-relevant color metric.

### 4.4. Limitations and Future Work

Although SAM2 is widely presented as an interactive, prompt-based segmentary, its core value in our application is that it is a pretrained, prompt foundation model whose representations can be fine-tuned for fully automated segmentation. In this study, we froze the prompt encoder and fine-tuned the image encoder and the mask decoder only, thereby removing any dependency on the user input at inference while benefiting from SAM2’s generalization and boundary-aware features. This design allows us to (i) leverage data-efficient transfer on a moderate-sized, single-domain dataset; (ii) isolate the effect of variant size (tiny, small, base plus, large) under a uniform pipeline; and (iii) preserve compatibility with future interactive corrections if required in clinical settings. The resulting models achieved a high DSC/IoU, a low normalized HD95, and ΔE_00_ distributions near the JND threshold, indicating that SAM2’s pretrained priors remain advantageous even in a non-interactive workflow.

While the dataset was annotated by a board-certified prosthodontist and comprised 500 images, it remains relatively small according to deep learning research. Larger and more diverse datasets—especially including variations in lighting, ethnicity, and device type—would validate the generalizability further. Furthermore, clinical trials evaluating the impact of AI-assisted shade matching on actual patient outcomes would provide real-world validation of the system’s utility.

In this study, we used UNet as a widely adopted baseline but did not include the more modern nnU-Net. The primary rationale was to maintain identical hyperparameters for a fair comparison with SAM2. Because nnU-Net is a self-configuring framework that automatically adapts the network depth and width, patch size, preprocessing and augmentation, loss weighting, cross-validation, ensembling, and test-time procedures to each dataset, it is difficult to disentangle whether performance differences arise due to the model capacity/architecture or the pipeline’s auto-configuration. By contrast, UNet has a fixed two-dimensional encoder–decoder design with high reproducibility, which allowed us to match and control key training settings—the optimizer, learning rate, scheduler, input resolution, loss functions, and augmentations—to those used for SAM2. We therefore adopted UNet as the baseline most aligned with our goal of isolating the effect of model scale among SAM2 variants under the same training recipe. Although nnU-Net is known to deliver a strong performance on many public benchmarks, direct head-to-head comparisons remain an important area for future investigation.

As further considerations, future work will be needed to include direct comparisons with graph-based vision backbones (e.g., MedSegViG) [29] and the investigation of explicit margin-aware objectives motivated by 3D trim-line frameworks [30], adapted to 2D intraoral photography.

### 4.5. The Computational Cost, Inference Time, and Deployment Feasibility

The exact throughput and memory usage depend on the hardware, software stack, and input resolution, but several stable patterns guide deployment choices. The UNet baseline has the smallest computational footprint and runs reliably on commodity GPUs and even modern CPUs, which makes it suitable for resource-constrained settings [18]. By contrast, the SAM2 variants require progressively more compute and memory as the model size increases: tiny is practical on mid-range GPUs (and some high-end integrated GPUs), small provides a strong balance between accuracy and latency on a single workstation-class GPU, and base plus and large benefit from higher-memory GPUs or server environments [10,12].

Because runtime and memory are strongly hardware- and resolution-dependent, we present a qualitative analysis here; a controlled, hardware-matched benchmarking study remains an important direction for future investigation.

## 5. Conclusions

Within a single dataset and training protocol, fine-tuned SAM2 variants and a UNet baseline achieved a high spatial overlap and reliable boundary alignment for simultaneous segmentation of the teeth and shade guides in intraoral photographs. SAM2-large obtained the highest average accuracy, while the smaller SAM2 models remained competitive at a lower computational cost.

Analyses of the color uniformity and perceptual color difference based on the mean–full-mask ΔE_00_ indicated low variability and small predicted-to-reference differences on average, suggesting that the segmented regions preserve chromatic properties relevant to shade assessments under the present acquisition conditions. These results should be interpreted as evidence of technical feasibility rather than proof of clinical equivalence since the perceptual thresholds and absolute color errors can depend on the illumination, device characteristics, and downstream workflow choices.

This study is limited by the use of a single-center dataset, a single convolutional baseline (UNet), and the absence of external validation or prospective evaluation in clinical settings. Accordingly, we do not claim clinical readiness or superiority over state-of-the-art task-specific methods. Instead, our findings provide a benchmark of the within-dataset performance across SAM2 scales and a convolutional reference that can guide subsequent work.

## Figures and Tables

**Table 1:** Architectural characteristics of UNet and SAM2 model variants.

Model Variant	Backbone	Number of Parameters	Input Resolution	Recommended Use Case
UNet	CNN encoder–decoder	~40 million	512 × 512 (configurable)	Classical baseline; fast training/inference; robust with limited data
SAM2-tiny	ViT-Tiny	~5 million	256 × 256	Lightweight applications, mobile deployment
SAM2-small	ViT-Small	~30 million	512 × 512	Real-time inference with efficiency–accuracy balance
SAM2-base+	ViT-Base Plus	~90 million	1024 × 1024	Balanced trade-off between performance and cost
SAM2-large	ViT-Large	~300 million	1024 × 1024 or higher	High-precision medical or scientific segmentation

## Data Availability

The datasets are not publicly available due to their potential use in a commercial application. Requests to access the datasets should be directed to Ki-Sun Lee (kisuns@gmail.com).

## References

[1] Clary J.A., Ontiveros J.C., Cron S.G., Paravina R.D. (2016). Influence of light source, polarization, education, and training on shade matching quality. J. Prosthet. Dent..

[2] Igiel C., Lehmann K.M., Ghinea R., Weyhrauch M., Hangx Y., Scheller H., Paravina R.D. (2017). Reliability of visual and instrumental color matching. J. Esthet. Restor. Dent..

[3] Rodrigues S., Shetty S.R., Prithviraj D. (2012). An evaluation of shade differences between natural anterior teeth in different age groups and gender using commercially available shade guides. J. Indian Prosthodont. Soc..

[4] Pohlen B., Hawlina M., Šober K., Kopač I. (2016). Tooth Shade-Matching Ability Between Groups of Students with Different Color Knowledge. Int. J. Prosthodont..

[5] Jouhar R. (2022). Comparison of Shade Matching ability among Dental students under different lighting conditions: A cross-sectional study. Int. J. Environ. Res. Public Health.

[6] Gurrea J., Gurrea M., Bruguera A., Sampaio C.S., Janal M., Bonfante E., Coelho P.G., Hirata R. (2016). Evaluation of Dental Shade Guide Variability Using Cross-Polarized Photography. Int. J. Periodontics Restor. Dent..

[7] Özat P., Tuncel İ., Eroğlu E. (2013). Repeatability and reliability of human eye in visual shade selection. J. Oral Rehabil..

[8] AlSaleh S., Labban M., AlHariri M., Tashkandi E. (2012). Evaluation of self shade matching ability of dental students using visual and instrumental means. J. Dent..

[9] Geetha A.S., Hussain M. (2024). From SAM to SAM 2: Exploring Improvements in Meta’s Segment Anything Model. arXiv.

[10] Ravi N., Gabeur V., Hu Y.-T., Hu R., Ryali C., Ma T., Khedr H., Rädle R., Rolland C., Gustafson L. (2024). Sam 2: Segment anything in images and videos. arXiv.

[11] Jiaxing Z., Hao T. (2025). SAM2 for Image and Video Segmentation: A Comprehensive Survey. arXiv.

[12] Kirillov A., Mintun E., Ravi N., Mao H., Rolland C., Gustafson L., Xiao T., Whitehead S., Berg A.C., Lo W.-Y. Segment anything. Proceedings of the 2023 IEEE/CVF International Conference on Computer Vision (ICCV).

[13] Ma J., He Y., Li F., Han L., You C., Wang B. (2024). Segment anything in medical images. Nat. Commun..

[14] Mazurowski M.A., Dong H., Gu H., Yang J., Konz N., Zhang Y. (2023). Segment anything model for medical image analysis: An experimental study. Med. Image Anal..

[15] Koch T.L., Perslev M., Igel C., Brandt S.S. Accurate segmentation of dental panoramic radiographs with U-Nets. Proceedings of the 2019 IEEE 16th International Symposium on Biomedical Imaging (ISBI 2019).

[16] Oktay O., Schlemper J., Folgoc L. (2018). Attention U-Net: Learning where to look for the pancreas. arXiv.

[17] Litjens G., Kooi T., Bejnordi B.E., Setio A.A.A., Ciompi F., Ghafoorian M., Van Der Laak J.A., Van Ginneken B., Sánchez C.I. (2017). A survey on deep learning in medical image analysis. Med. Image Anal..

[18] Ronneberger O., Fischer P., Brox T. U-net: Convolutional networks for biomedical image segmentation. Proceedings of the International Conference on Medical image computing and computer-assisted intervention.

[19] Ma J., Yang Z., Kim S., Chen B., Baharoon M., Fallahpour A., Asakereh R., Lyu H., Wang B. (2025). Medsam2: Segment anything in 3d medical images and videos. arXiv.

[20] Sengupta S., Chakrabarty S., Soni R. Is SAM 2 better than SAM in medical image segmentation?. Proceedings of the Medical Imaging 2025: Image Processing.

[21] Taha A.A., Hanbury A. (2015). Metrics for evaluating 3D medical image segmentation: Analysis, selection, and tool. BMC Med. Imaging.

[22] Reinke A., Tizabi M.D., Sudre C.H., Eisenmann M., Rädsch T., Baumgartner M., Acion L., Antonelli M., Arbel T., Bakas S. (2021). Common limitations of image processing metrics: A picture story. arXiv.

[23] Kusayanagi T., Maegawa S., Terauchi S., Hashimoto W., Kaneda S. (2023). A smartphone application for personalized tooth shade determination. Diagnostics.

[24] Luo M.R., Cui G., Rigg B. (2001). The development of the CIE 2000 colour-difference formula: CIEDE2000. Color Research & Application: Endorsed by Inter-Society Color Council, The Colour Group (Great Britain), Canadian Society for Color, Color Science Association of Japan, Dutch Society for the Study of Color, The Swedish Colour Centre Foundation, Colour Society of Australia, Centre Français de la Couleur.

[25] Paravina R.D., Ghinea R., Herrera L.J., Bona A.D., Igiel C., Linninger M., Sakai M., Takahashi H., Tashkandi E., Mar Perez M.d. (2015). Color difference thresholds in dentistry. J. Esthet. Restor. Dent..

[26] Huang Y., Yang X., Liu L., Zhou H., Chang A., Zhou X., Chen R., Yu J., Chen J., Chen C. (2024). Segment anything model for medical images?. Med. Image Anal..

[27] Sirintawat N., Leelaratrungruang T., Poovarodom P., Kiattavorncharoen S., Amornsettachai P. (2021). The accuracy and reliability of tooth shade selection using different instrumental techniques: An in vitro study. Sensors.

[28] Sharma G., Wu W., Dalal E.N. (2005). The CIEDE2000 color-difference formula: Implementation notes, supplementary test data, and mathematical observations. Color Research & Application: Endorsed by Inter-Society Color Council, The Colour Group (Great Britain), Canadian Society for Color, Color Science Association of Japan, Dutch Society for the Study of Color, The Swedish Colour Centre Foundation, Colour Society of Australia, Centre Français de la Couleur.

[29] Li X., Chen G., Wu Y., Yang J., Zhou T., Zhou Y., Zhu W. MedSegViG: Medical Image Segmentation with a Vision Graph Neural Network. Proceedings of the 2024 IEEE International Conference on Bioinformatics and Biomedicine (BIBM).

[30] Chen G., Qin J., Amor B.B., Zhou W., Dai H., Zhou T., Huang H., Shao L. (2023). Automatic detection of tooth-gingiva trim lines on Dental Surfaces. IEEE Trans. Med. Imaging.

